# Frequency and type of toenail tumors in the dromedary camel

**Published:** 2013-06-28

**Authors:** M.I. Siddiqui, S.A. Al-Kubati, M.N. Telfah, J. Rashid, S. Hashmi

**Affiliations:** *Central Veterinary Hospital, Al-Wathba, Abu Dhabi, United Arab Emirates*

**Keywords:** Camels, Squamous cell carcinoma, Toenails, Tumor

## Abstract

A total of 275 dromedary camels (16 males and 259 females) of local *“Arabiyat”* breed suffering from different types and degrees of severity of toenail tumors were surgically treated. Histopathological examination of the tissue samples removed from 50 tumor-like growths (2 males and 48 females) revealed three types of tumors; squamous cell carcinoma (70%), spiny keratoderma (22%) and fibroma (8%). An increased incidence of tumors was recorded in the medial when compared to the lateral toenails in both sexes. In females, the incidence in the medial toenails was 90/259 (34.75%) and 71/259 (27.41%) in the right and left forelimbs respectively when compared to the lateral toenails which was 25/259 (9.65%) and 5/259 (1.93%) for the respective right and left forelimbs. In the hind limbs, this ratio was 29/259 (11.20%) and 20/259 (7.72%) for right and left medial toenails respectively, whereas it was 17/259 (6.56%) and 2/259 (0.77%) for the right and left lateral toenails respectively. Similar to the observations in female camels, male camels also showed a higher incidence of these tumors in the medial when compared to the lateral toenails in both fore and hind limbs. Based on these findings, we conclude that in the dromedary camels, the medial toenails of the fore limbs are most commonly affected with tumors; with the most common tumor being the squamous cell carcinoma.

## Introduction

Camelids are modified digitigrades with a small, non-weight bearing nail which is similar to a human nail and is located at the extremity of each digit. The nail is closely attached to the third phalanx via the corium. The pectoral limb is the weight bearing axis of the body and there is a slight difference in the anatomical configuration between the lateral and medial metacarpal bones and the digits in camels.

The proximal articular surface of the third metacarpal bone is slightly higher than that of the fourth metacarpal and its mediopalmar facet articulates only with the second carpal bone.

The distal articular surface of the third metacarpal bone is also at a slightly higher plane than that of the fourth metacarpal bone. The first and second lateral phalanges are ~2-3 mm longer than their medial counter parts (Smuts and Bezuidenhout, 1987).

This anatomical configuration results in a slight but noticeable lateral deviation of the forelimbs extending from the knee to the foot. This configuration may subject the medial digit to increased pressure when compared to the lateral digit, rendering the former more prone and vulnerable to pressure-related injuries leading to abnormal growth lesions (Siddiqui and Telfah, 2010).

Although the hind limbs in the camel are not a part of the weight bearing axis of the body, the anatomical configuration of the metatarsal bones and the digits resembles that of the corresponding bones of the fore limbs, as the first and second lateral phalanges are 1-2 mm longer than their medial counter parts (Smuts and Bezuidenhout, 1987). Previous reports on tumors in camelids include squamous cell carcinoma of the skin in a llama (Rogers *et al.*, 1997), squamous cell carcinoma of the foot and chondrosarcoma of the left carpal joint in camels (Gahlot *et al.*, 1995; Janardhan *et al.*, 2011).

A general report of neoplasia in camels (Tageldin and Omer, 1986; Singh *et al.*, 1991; Ramadan, 2004), cutaneous melanocytoma in a llama (Radi *et al.*, 2005) and a basal cell carcinoma in a dromedary camel (Al-Hizab *et al.*, 2007) have been previously published.

In our experience, toenail tumors appear to be the most common type of cutaneous tumors observed in dromedary camels. The tumor initially involves the toenail and as the time progresses, the tumor advances proximally to a variable degree to further involve the digit and the sole. In the majority of the cases, the lesion affects the medial toenails/digits of the forelimbs (Siddiqui and Telfah, 2010).

The development of the tumor is progressive and the animal is often presented for treatment when the tumor is at an advanced stage. Onychia (inflammation of the corium beneath the nail) and paronychia (inflammation of the tissue at the margins of the nail) in camelids can result from either contusions or lacerations of the nail. Neglected toenail trimming in such cases may allow the infection to migrate dorsally under the nail (Murray and Bravo, 2010).

If further neglected, such conditions can exacerbate and act aggressively leading to the involvement of the soft tissue beyond the toenail.

A squamous cell carcinoma typically originates from the skin around the toenail and commonly affects the surrounding bone and soft tissue. Because this tumor spreads slowly, it can often be visualized before it spreads to other areas of the body.

The most common symptom at this stage is a swollen toe with or without lameness. Radical surgical excision of the mass along with the toenail is most likely the only treatment that can consistently provide relief to the animal and also potentially avoid metastasis of the tumor to other sites (Murray and Bravo, 2010).

Squamous cell carcinoma is also reported in the dogs. Similar to that observed in the camel, the tumor in the dogs affects only one toe and is noticeable grossly as solid, raised skin mass over the affected toe. However, over time the tumor expands and loses its original solid mass-like appearance as the tissue within the mass dies. Eventually the tumor ulcerates and subsequently extends proximally to involve the digit (Goldschmidt and Shofer, 1998).

In our experience, the toenail tumor in the camel follows almost a similar course as that reported in the dog.

Because the toenail/digit tumors in the dromedary camel are very frequent in the emirate of Abu Dhabi, United Arab Emirates, we sought to study this widespread condition in depth including an investigation of the incidence and nature of this endemic disease.

## Materials and Methods

The study included 275 cases (16 male and 259 female camels) that displayed growth around the soft tissues above the nail. These cases were reported to the Central Veterinary Hospital, Al-Wathba, Abu Dhabi, United Arab Emirates during a period of 8 years (January, 2004 to December, 2011). The cases were categorized on the basis of sex, the limb and toenail involved (Table 1).

**Table 1:** Prevalence of toenail/digit tumors in the dromedary camels (recorded during Jan. 2004 to Dec. 2011), brought for treatment to the Central Veterinary Hospital, Al-Wathba, Abu Dhabi, United Arab Emirates.

	Male				Female			
	R	L	R	L	R	L	R	L
	F	F	H	H	F	F	H	H
MT/DGT	4	2	3	4	90	71	29	20
LT/DGT	2	-	1	-	25	5	17	2
Subtotal	6	2	4	4	115	76	46	22
Total	16				259			

RF = Right fore-limb; LF = Left fore-limb; RH = Right hind-limb; LH = Left hind-limb; MT/DGT = Medial Toe/Digit; LT/DGT = Lateral Toe/Digit.

**Fig. 1:**
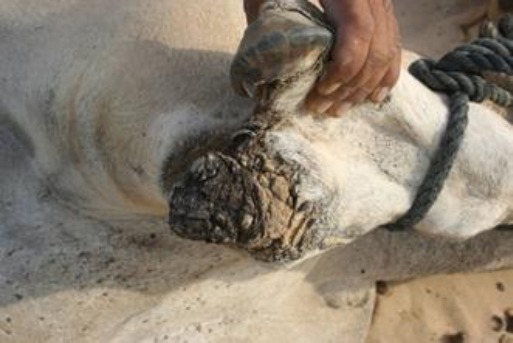
A spiny keratoderma involving the medial toenail in the right fore limb of a female dromedary camel. The tumor mass was not painful, was hard on palpation, and also not aggressive in its appearance.

**Fig. 2:**
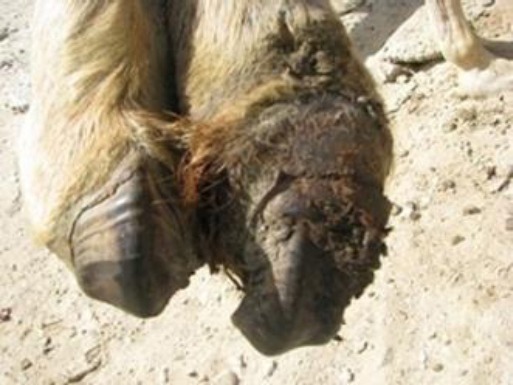
A fibroma involving almost half of the medial digit in the left fore limb of a female dromedary camel. The mass was slightly moist, hard and painful upon palpation.

**Fig. 3:**
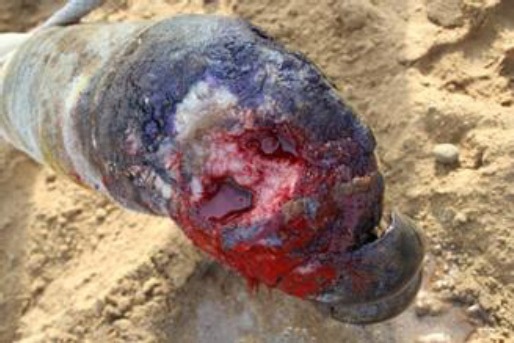
A squamous cell carcinama involving more than half of the medial digit in a female dromedary camel. The mass was aggressive, fragile, quite painful and bled easily on manipulation.

Our study shows that the growth involved the toenail only (Figure 1) or almost half of the digit (Figure 2) or more than half or full digit (Figure 3).

### Surgical Intervention

In each case, the severity and extent of involvement of the digit was carefully evaluated in order to decide the most appropriate surgical procedure to provide relief to the suffering animal.

**Fig. 4:**
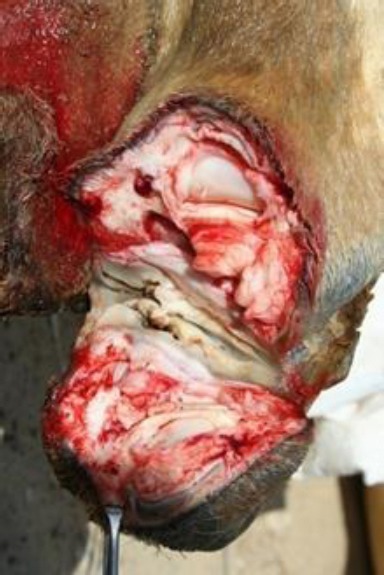
Disarticulation at the distal interphalangeal joint.

**Fig. 5:**
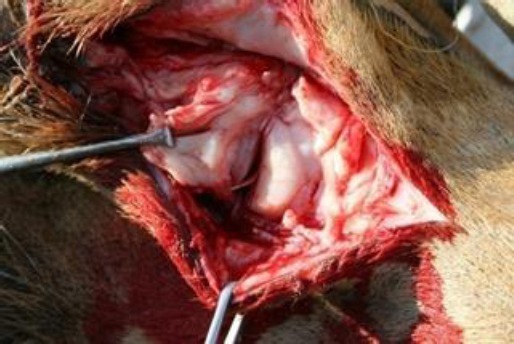
Disarticulation at the proximal interphalangeal joint.

When only the toenail was involved, the third phalanx along with the toenail was removed by disarticulation at the distal interphalangeal joint (Figure 4). In case of involvement of half of the digit, disarticulation was performed at the proximal interphalangeal joint (Figure 5).

**Fig. 6:**
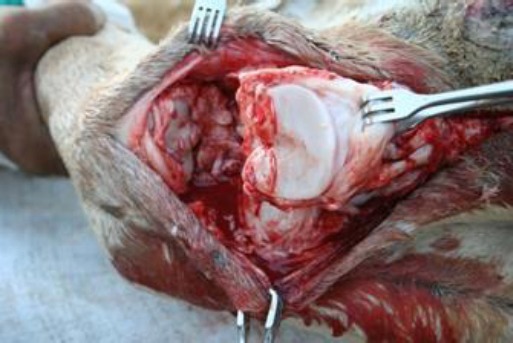
Disarticulation at the metacarpo-phalangeal joint.

When the tumor involved more than half or the entire digit; unilateral disarticulation of the digit was performed at the metacarpo-phalangeal or metatarso-phalangeal joint with excision of the diseased tissue while preserving the normal sole (Figure 6).

This procedure resulted in restoring the maximum weight bearing surface of the foot, thus enabling the animal to attain a near-normal gait soon after the recovery.

In all surgical interventions, a strict adherence to the goals of cancer surgery was assured: viz; a complete excision of the cancerous tissue along with the surrounding healthy tissue to minimize chances of tumor recurrence and to prevent an adverse metastasis.

### Histopathological Evaluation

A total of 275 cases were operated for tumor-like lesions. Tissue samples from 50 cases (2 males and 48 females) showing different growth patterns were collected for histological examination. Representative sections from each tumor tissue were fixed in 10% neutral buffered formalin and were submitted to the Pathology Laboratory of Al-Noor Hospital, Abu Dhabi for a detailed histopathological evaluation.

### Statistical analysis

The data was analysed using the chi-square test. The criterion for statistical significance was set at *P* < 0.05.

## Results

### Healing of the operative wounds

None of the operative wounds healed through first intention. The wounds in different animals required variable time periods to heal ranging from 2 to 4 months. Recurrence of the tumor was not observed in any of the operated cases.

### Most common toe involved

An increased incidence of the tumors was recorded in the medial toenails/digits of the fore and hind limbs in both sexes (Tables 2 and 3). In the hind limbs of the females, the incidence of the tumors was lower, but the involvement of the medial toenail was greater than the lateral toenail.

**Table 2:** Incidence of toenail tumors in male camels.

Toenail/Digit Involved	No. of animals with tumors	Total number of animals	%	*P* value
RFM	4	16	25	*P*=0.173
RFL	2	16	12.50	*P*=0.173
LFM	2	16	12.50	*P*=0.173
LFL	-	16	0.00	*P*=0.173
RHM	3	16	18.75	*P*=0.173
RHL	1	16	06.25	*P*=0.173
LHM	4	16	25.00	*P*=0.173
LHL	-	16	0.00	*P*=0.173

RFM = Right fore medial; RFL = Right fore lateral; LFM = Left fore medial; LFL = Left fore lateral; RHM = Right hind medial; RHL = Right hind lateral; LHM = Left hind medial; LHL = Left hind lateral. Chi-square value = 10.285. *P*-Value = 0.173 (*P*> 0.05). Inference: There is no significant statistical difference between involvement of fore and hind limbs or the medial and lateral toenails in male camels.

**Table 3:** Incidence of toenail tumors in female camels.

Toenail/Digit Involved	No. of animals with tumors	Total number of animals	%	*P* value
RFM	90	259	34.75	*P*=0.00001
RFL	25	259	09.65	*P*=0.00001
LFM	71	259	27.41	*P*=0.00001
LFL	5	259	01.93	*P*=0.00001
RHM	29	259	11.20	*P*=0.00001
RHL	17	259	06.56	*P*=0.00001
LHM	20	259	07.72	*P*=0.00001
LHL	2	259	00.77	*P*=0.00001

RFM = Right fore medial; RFL = Right fore lateral; LFM = Left fore medial; LFL = Left fore lateral; RHM = Right hind medial; RHL = Right hind lateral; LHM = Left hind medial; LHL = Left hind lateral. Chi-square value = 244.9818. *P*-Value = 0.00001 (*P*< 0.05). Inference: There is a significant statistical difference between involvement of fore and hind limbs and the medial and lateral toenails in female camels (fore limbs are more prone to toenail tumors than the hind limbs. Likewise the medial toenails are more prone to develop tumors than the lateral toenails).

### Histopathological findings

Histopathological examination revealed that the toenail tumors included one of three following types; Squamous Cell Carcinoma, Spiny Keratoderma or fibroma. The incidence of three different types of toenail tumors (in 50 tissue samples collected) is shown in Table 4. Of all three tumor types, squamous cell carcinoma ranked first (70%) in its incidence. It was generally observed that clinically aggressive tumor lesions had more chances of being malignant.

**Table 4:** Types of toenail/digit tumor lesions.

Type of Tumor Lesion	Number Recorded	Total Number	%	*P* value
Squamous Cell Carcinom	35	50	70	*P*=0.00001
Spiny Keratoderm	11	50	22	*P*=0.00001
Fibrom	4	50	8	*P*=0.00001

Chi-square value = 47.58. *P*-Value = 0.00001 (*P*< 0.05). Inference: There is a significant statistical difference among different types of toenail tumors with squamous cell carcinoma being the most commonly observed tumor.

Squamous Cell Carcinoma lesions consisted of moderate to well-differentiated sheets of small and average sized squamous cells.

Frequent clusters of anaplastic squamous epithelial cells with central keratin pearls were also observed. The nuclei were larger in size and had an average number of 2-3 nucleoli per nucleus.

**Fig. 7:**
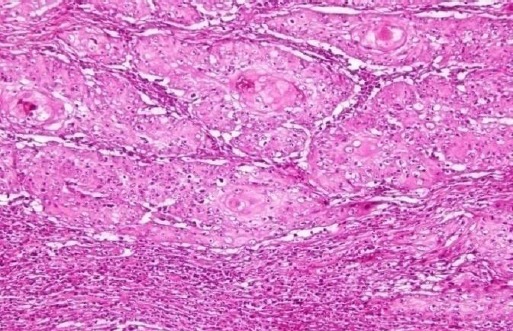
Squamous Cell Carcinoma (H&E Staining, ×40).

The lesions showed frequent angulations with desmoplastic stroma and polymorphic inflammatory infiltrate. No vascular or perineural invasion was observed (Figure 7).

**Fig. 8:**
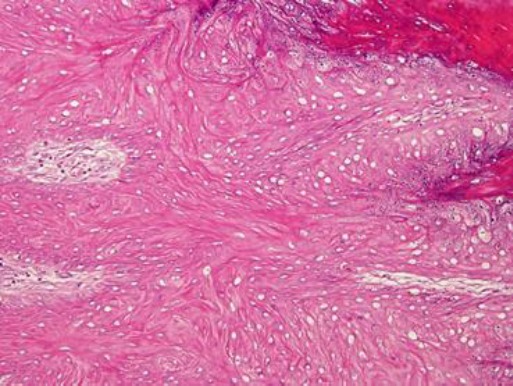
Spiny Keratoderma (H&E Staining, ×40).

Spiny Keratoderma lesions had thick spiny keratotic projections consisting of a well-defined compact column of parakeratotic horn material directly continuous with granular layer. Abrupt transition to orthokeratotic corneal layer at the margins of parakeratotic column was also observed. No dyskeratotic or vacuolated keratinocytes were observed in the underlying epidermis. Furthermore, no malignancy was observed (Figure 8).

**Fig. 9:**
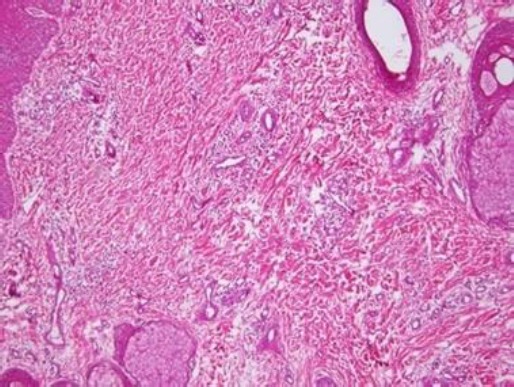
Fibroma (H&E Staining, ×40).

Fibroma lesions had thick skin tissue showing evident areas of acanthosis and spiny hyperkeratosis. There was abnormal deposition of fibrocollagenous material involving up to the subcutis with wavy fibrous tissue infiltrating and dissecting the underlying structures including the pileosebaceous units, muscles and sweat glands. Whorl appearance was also noticed in some of the areas (Figure 9).

## Discussion

None of the surgical wounds healed through first intention. Considering the anatomic location of the lesions and the type of surgical manipulations required, first intention healing of the wounds in such cases is not likely (Murray and Bravo, 2010; Siddiqui and Telfah, 2010). Complete surgical excision of the tumor along with the surrounding healthy tissue most likely minimized the probability of tumor recurrence (Tageldin and Omer, 1986; Gahlot *et al.*, 1995).

The fact that fore limbs are the weight bearing axis of the body along with their unique anatomic configuration from the knee to the foot may help explain an increased incidence of tumors in the medial toes of the forelimbs (Smuts and Bezuidenhout, 1987; Siddiqui and Telfah, 2010).

In our opinion, the possible reason for the lower incidence of tumors in the hind limbs is that these are not a part of weight bearing axis of the body and therefore, are not subjected to higher pressure under the body weight of the animal. However, the anatomical configuration of the metatarsal bones and the digits resembles that of the fore limbs. This may be a plausible reason for an increased incidence of tumors in the medial when compared to the lateral toenails/digits in the hind limbs (Smuts and Bezuidenhout, 1987).

The significant observation of our study that clinically aggressive tumor lesions have increased chances of being malignant agrees well with the previous reports (Tageldin and Omer, 1986; Gahlot *et al.*, 1995; Rogers *et al.*, 1997; Al-Hizab *et al.*, 2007; Janardhan *et al.*, 2011).

In summary, salient findings from our study reveal that the medial toenails of the fore limbs are frequently prone to squamous cell carcinomas and that a timely surgical excision of the tumorous mass completely alleviates this afflictive condition in camels.
